# The Mediating Role of Depression in the Association Between Death Anxiety and Quality of Life in Elderly Prostate Cancer Patients

**DOI:** 10.62641/aep.v53i4.1921

**Published:** 2025-08-05

**Authors:** Haifeng Song, Fengyi He, Xiaoling Zhang

**Affiliations:** ^1^Department of Urology, Lu'an Hospital of Anhui Medical University, 237005 Lu'an, Anhui, China

**Keywords:** prostate cancer, depression, death anxiety, quality of life, mediating effects

## Abstract

**Background:**

A decreased quality of life is commonly observed in elderly patients with prostate cancer (PCa), and psychological changes in these patients require particular attention. This study aimed to investigate factors influencing depression, death anxiety, and quality of life in elderly PCa patients and to explore the mediating role of depression in the relationship between death anxiety and quality of life.

**Methods:**

A total of 120 valid questionnaires from PCa patients at Lu'an Hospital of Anhui Medical University were collected between October 2021 and July 2024. The Templer Death Anxiety Scale (T-DAS), Zung Self-Rating Depression Scale (SDS), and Functional Assessment of Cancer Therapy for PCa (FACT-P) were used to assess death anxiety, depression, and quality of life, respectively. Influencing factors and the mediating role of depression between death anxiety and quality of life were analyzed.

**Results:**

(1) Patients had a mean SDS score of 56.93 ± 4.47, T-DAS score of 44.83 ± 7.18 and FACT-P score of 103.52 ± 6.22; (2) Univariate analyses showed that patients' depression levels were associated with occupational status, average monthly income, length of illness, primary caregiver, and number of chronic illnesses (*p* < 0.05); death anxiety levels were associated with age, length of illness, primary caregiver, and number of chronic illnesses (*p* < 0.05); and quality of life was associated with age, BMI, and number of primary caregivers and chronic illnesses (*p* < 0.05); (3) Correlation analysis showed that depression was negatively correlated with quality of life (*ρ* = –0.360, *p* < 0.001), death anxiety was negatively correlated with quality of life (*ρ* = –0.456, *p* < 0.001), and death anxiety was positively correlated with depression (*ρ* = 0.493, *p* < 0.001); (4) Death anxiety had a significant negative effect on quality of life, with a direct effect of –0.262 and a total effect of –0.429. Depression significantly mediated the relationship between death anxiety and quality of life, with a mediating effect of –0.167 (95% CI: –0.331 to –0.045).

**Conclusion:**

Depressive symptoms and death anxiety are prevalent among elderly PCa patients. Death anxiety directly impacts the quality of life of patients and also mediates an indirect effect through depression, further reducing patients' quality of life.

## 1. Introduction

As lifestyles and dietary habits change, the incidence of prostate cancer (PCa) among elderly men has been steadily increasing. This malignancy has become the most prevalent cancer in the urinary and reproductive systems among males, significantly affecting the quality of life and life expectancy of this population [1, 2]. PCa typically has an insidious onset, with slow tumor growth over several years, and most patients remain asymptomatic during the early stages [3]. However, as the tumor grows, enlargement of the prostate gland and pressure on the urinary tract, or even in cases of bone metastasis due to the spread of the cancer cells may result in symptoms such as frequent urination, urgency of urination, painful urination, hematuria, difficulty urination, or urinary retention [4]. Additionally, it can lead to erectile dysfunction, difficulty in ejaculation, or reduced semen volume. In advanced stages, when metastasis has occurred, patients may experience chronic bone pain, rectal pressure, or constipation [5]. Diagnosis is typically confirmed through clinical examination, including digital rectal examination, serological testing, ultrasound, magnetic resonance imaging (MRI), and prostate tissue biopsy. Treatment generally falls into two categories: palliative care and radical resection. Palliative care, also known as “watchful waiting”, is primarily for patients with advanced PCa or those with low-risk PCa but limited life expectancy. In contrast, radical prostatectomy remains the most effective treatment for localized or locally advanced PCa, significantly improving survival rates. However, postoperative complications such as urethral stricture, urinary incontinence, urinary fistula, and deep vein thrombosis are common [6, 7].

In addition to the disease symptoms and physical side effects of treatment, patients with PCa face a variety of psychological, social, and multidisciplinary challenges. Advances in clinical diagnosis and treatment have extended the lives of many patients with PCa. However, researchers have discovered that a significant proportion of these patients experience severe side effects, including depression, as a result of extended survival and the influence of modern medical models and health ideas. These side effects can lead to symptoms such as low mood, insomnia, fatigue, and even despair and are considered risk factors for suicide-related deaths among cancer patients [8, 9]. A meta-analysis [10] further demonstrated that depression increases the risk of mortality in cancer patients by 39%.

Previous research has shown that death anxiety is a conscious or unconscious psychological state that arises when individuals face the threat of death, triggering defense mechanisms [11]. It has been recognized as a nursing diagnosis by the North American Nursing Diagnosis Association, and death anxiety has been shown to affect the quality of life. Studies have shown that more than 43% of patients with advanced cancer report moderate to severe death anxiety, significantly impacting their physical and mental health, prognostic outcomes, and overall quality of life [11, 12]. A longitudinal study on PCa patients found that most treatments lead to deterioration in quality of life, especially within the first year of treatment, with variations depending on the treatment type [13, 14].

The primary objective of this study was to investigate factors influencing depression, death anxiety, and quality of life in elderly PCa patients, as well as to explore the mediating role of depression in the relationship between death anxiety and quality of life in this patient population.

## 2. Methods

### 2.1 Sample Size and Study Design

According to the sample size calculation formula: n = Z_a_/2(1-P)/ε^2^*p*, where a = 0.05, Z_a_/2 = 1.96, ε = 0.25, *p* = 0.40, the required sample size was calculated to be 98 cases. Considering a 20% questionnaire loss rate, the final selected sample size was determined to be 130 cases.

### 2.2 Clinical Data

**Fig. 1.**
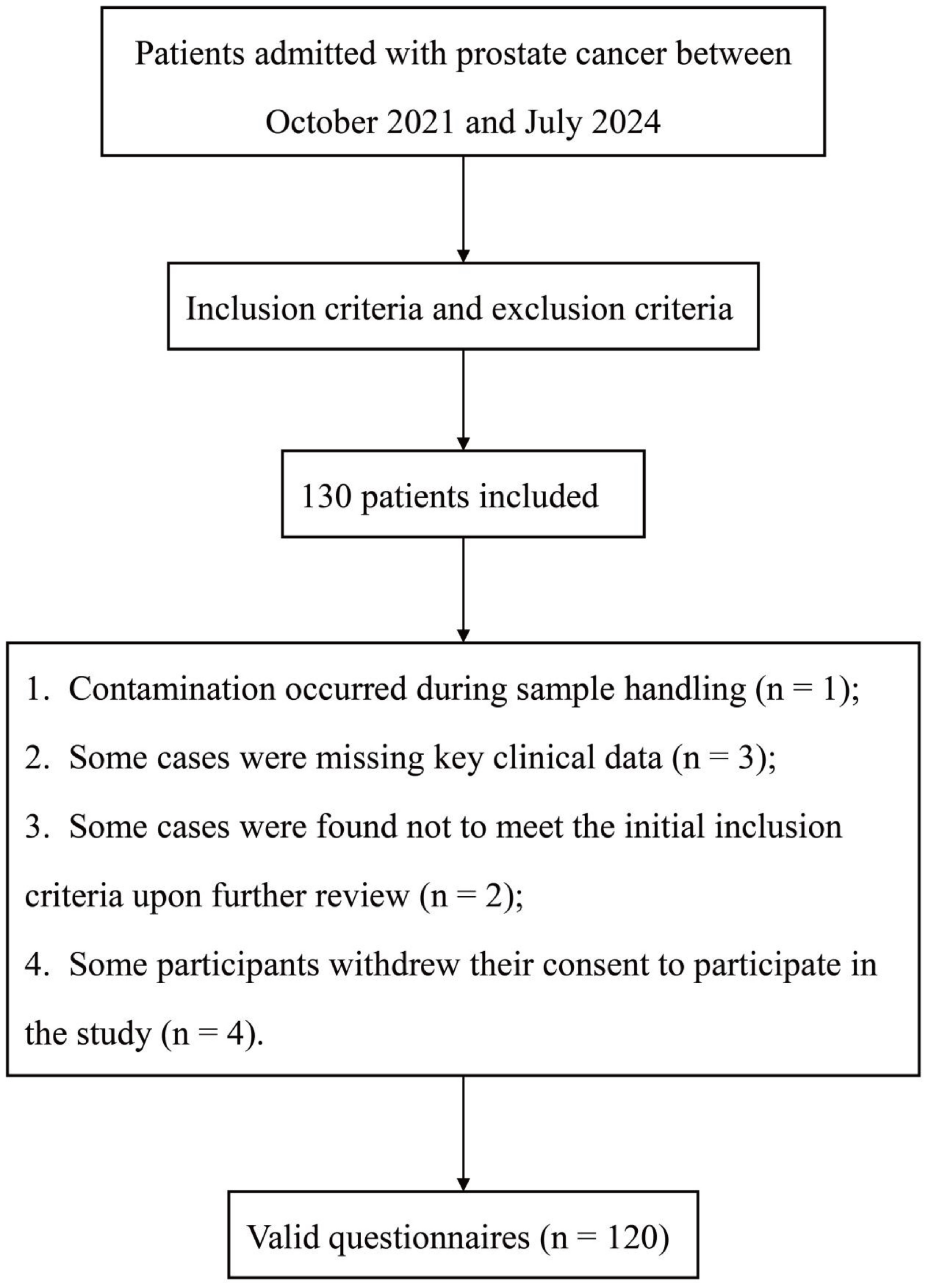
**The flow chart of study design**.

PCa patients admitted to the Lu’an Hospital of Anhui Medical University between October 2021 and July 2024 were selected for this study. The inclusion criteria were: (1) Patients diagnosed with PCa by rectal digital examination, laboratory tests, transrectal ultrasound, and magnetic resonance imaging, confirmed by histopathological examination [15]; (2) Patients who have previously undergone treatment (including endocrine therapy, surgery, or radiation therapy); (3) Patients with basic knowledge of the disease; (4) Patients with clear consciousness and unimpaired communication abilities; (5) Patients who were informed and provided written consent to participate in the study. The exclusion criteria included: (1) Patients with other malignant tumors in addition to PCa; (2) Patients with difficulty in expressing their wishes, such as those with impaired consciousness or dementia; (3) Individuals diagnosed with depression, anxiety disorders, or other severe psychiatric conditions prior to the diagnosis of PCa; (4) Patients with significant depressive or anxiety symptoms at the time of enrollment; (5) Patients or their family members who refused to participate in the study. A total of 130 questionnaires were distributed, and 120 valid responses were collected, resulting in a valid response rate of 92.3% (Fig. 1). This study protocol was approved by the hospital ethics committee, and all participants provided informed consent (approval number: PJ2021-08-43).

### 2.3 Investigative Tools

(1) A self-designed general information questionnaire was used to collect patient demographics, including age, BMI, educational level, marital status, occupational status, average monthly household income, primary caregiver, number of chronic conditions, and length of hospital stay.

(2) Templer’s Death Anxiety Scale (T-DAS) was developed by Prof. Donald I. Templer in 1967 [16]. The scale consists of 15 items, with 9 positively scored and 6 negatively scored. The scale is based on the original two-factor theory and has been supported by numerous experimental studies. It has been translated and adapted into multiple languages and is widely recognized by experts in the field as the ‘gold standard’ for measuring death anxiety.

(3) Zung self-rating Depression Scale (SDS): The SDS includes all current diagnostic criteria for depressive disorders, according to the American Psychological Association. It consists of 20 items assessing the frequency of depressive symptoms over the past two weeks, scored as follows: 1 (none or a little of the time), 2 (some of the time), 3 (a good portion of the time), and 4 (all or almost all of the time). The total scores range from 20 to 80, with higher scores indicating more severe depression. The items are categorized into four subtypes: depressed mood (6 items), anhedonia (4 items), cognitive depression (4 items), and somatic depression (6 items). The average score for each dimension is calculated by dividing the total score for the dimension by the number of items. According to the established cutoff, patients with SDS scores of 53 and above are considered clinically depressed [17].

(4) Quality of life assessment: Quality of life was assessed using the Functional Assessment of Cancer Therapy-Prostrate (FACT-P) scale [18]. The scale consists of the Functional Assessment of Cancer Therapy (FACT-G) and the Prostrate Cancer subscale (PCS). The FACT-G consists of five dimensions, including physical status, social/family status, emotional status, living status and doctor-patient relationship, with a total of 29 entries; the PCS module consists of 12 entries, which can assess the PCa patients’ body mass, appetite, pain, dysuria, dyspareunia, defecation difficulties, and sexual function status. Each item is scored using a 5-point Likert scale ranging from 0 (not at all) to 4 (very much). The total score of the FACT-G module ranges from 0 to 116, while the PCS module ranges from 0 to 48, with higher scores indicating better quality of life [18]. The average score for each dimension is obtained by dividing the total score by the number of items.

### 2.4 Variable Categorization Methods

In this study, several continuous and ordinal variables were converted into categorical variables to facilitate analysis. The categorization methods for each variable are described as follows: Age was classified into two groups: below 75 years (coded as 0) and aged 75 years or above (coded as 1). BMI was dichotomized, with BMI of less than 24 kg/m^2^ coded as 0 and BMI of 24 kg/m^2^ or above coded as 1. Years of schooling were categorized into three levels: less than or equal to 6 years (coded as 0), 6 to 9 years (coded as 1), and more than 9 years (coded as 2). Marital status was dichotomized into two categories: having a spouse (coded as 0) and not having a spouse (coded as 1). Occupational status was classified into three categories: currently employed (coded as 0), retired (coded as 1), and others (coded as 2). Average monthly household income was divided into three categories: less than 3000 CNY (1 CNY ≈ 0.14 USD) (coded as 0), 3001 to 6000 CNY (coded as 1), and more than 6000 CNY (coded as 2). Duration of illness was categorized using 6 and 12 months as cutoffs: less than 6 months (coded as 0), 6–12 months (coded as 1), and 12 months or more (coded as 2). The primary caregiver was categorized into three groups: spouse (coded as 0), son or daughter (coded as 1), and others (coded as 2). The number of chronic diseases was divided into three categories: 0 (coded as 0), 1 (coded as 1), and 2 or more (coded as 2). Length of hospital stay was categorized using 15 days as the cutoff: less than 15 days (coded as 0) and 15 days or more (coded as 1).

### 2.5 Statistical Methods

All data were processed using SPSS version 22.0 (IBM Corporation, Armonk, NY, USA). Continuous variables included SDS, T-DAS, and FACT-P scores, while categorical variables included demographic and clinical characteristics such as occupational and marital status. Continuous data were first tested for normality. Data conforming to a normal distribution were expressed as mean ± standard deviation ($\bar{x}$ ± s). Independent samples *t*-tests and one-way analysis of variance (one-way ANOVA) were used to compare SDS, T-DAS, and FACT-P scores across groups stratified by demographic factors. Data that followed a normal or approximately normal distribution were analyzed using Spearman’s correlation, while data that did not meet normality assumptions were analyzed using Spearman’s correlation. The mediating role between depression, death anxiety, and quality of life was examined using the SPSS macro-programme Process 4.1. The mediating role of depression in the relationship between death anxiety and quality of life was tested by the Bootstrap method with 5000 resamples and 95% confidence intervals. A *p* < 0.05 was considered statistically significant.

## 3. Results

### 3.1 Demographic and Disease-related Information

The relevant demographic and disease-related information of PCa patients in this study included age, BMI, years of schooling, marital status, occupational status, duration of illness, primary caregiver, per capita monthly household income, number of chronic diseases, and length of hospital stay. The detailed analysis results are shown in Table 1.

**Table 1.** **Demographic and clinical characteristics of PCa patients**.

Variables		n	%
Age (years)			
	<75	83	69.17%
	≥75	37	30.83%
BMI (kg/m^2^)			
	<24	94	78.33%
	≥24	26	21.67%
Years of schooling			
	≤6 years	41	34.17%
	6–9 years	52	43.33%
	>9 years	27	22.50%
Marital status			
	With a spouse	100	83.33%
	Without a spouse	20	16.67%
	Occupational status		
	Employed	16	21.67%
	Retired	97	80.83%
	Other	7	5.83%
Average monthly household income (CNY)			
	<3000	26	21.67%
	3001–6000	83	69.17%
	>6000	11	9.17%
Duration of illness			
	<6 months	59	49.17%
	6–12 months	30	25.00%
	≥12 months	31	25.83%
Primary caregiver			
	Spouse	74	61.67%
	Son or daughter	31	25.83%
	Other	15	12.50%
Number of chronic diseases			
	0	5	4.17%
	1	51	42.50%
	≥2	64	53.33%
Length of hospital stay			
	<15 days	76	63.33%
	≥15 days	46	38.33%

Note: BMI, body mass index; CNY, Chinese Yuan (1 CNY ≈ 0.14 USD).

### 3.2 SDS, T-DAS, and FACT-P Scores in PCa Patients

As shown in Table 2, the mean total SDS and T-DAS scores were (56.93 ± 4.47) and (44.83 ± 7.18), respectively. The mean total FACT-P scale score in elderly PCa patients was (103.52 ± 6.22). Among the PACT-G module dimensions, the lowest mean score was observed in the mood state dimension (2.26 ± 0.45).

**Table 2.** **SDS, T-DAS, and FACT-P scores in PCa patients**.

Variables		Total Score ($\bar{x}$ ± s)	Average Score ($\bar{x}$ ± s)
SDS		56.93 ± 4.47	
	-Depressed mood	15.50 ± 2.95	2.58 ± 0.49
	-Anhedonia	12.65 ± 3.01	3.12 ± 0.66
	-Cognitive depression	14.47 ± 3.14	3.56 ± 0.63
	-Somatic depression	14.25 ± 2.57	2.36 ± 0.41
T-DAS		44.83 ± 7.18	
FACT-P		103.52 ± 6.22	
	-FACT-G overall status	76.78 ± 4.16	
	-Physical status	18.45 ± 2.34	2.52 ± 0.50
	-Social/family status	19.42 ± 2.33	2.78 ± 0.30
	-Emotional status	15.25 ± 1.85	2.26 ± 0.45
	-Living status	16.33 ± 2.13	2.35 ± 0.46
	-Doctor-patient relationship	7.33 ± 1.15	3.45 ± 0.50
	-PCS module	26.74 ± 4.53	

Note: SDS, Zung Self-Rating Depression Scale; T-DAS, Templer’s Death Anxiety Scale; FACT-P, Functional Assessment of Cancer Therapy-Prostate; FACT-G, Functional Assessment of Cancer Therapy-General; PCS, Prostate Cancer Subscale.

### 3.3 Status of SDS Scores and Influencing Factors in PCa Patients

The results indicated significant differences (*p* < 0.05) in total SDS scores across groups stratified by occupational status, average monthly household income, duration of illness, primary caregiver, and number of chronic diseases in PCa patients (Table 3). Spearman correlation analysis between continuous independent variables and total SDS scores showed that SDS scores of PCa patients were not significantly correlated with marital status (ρ = 0.008, *p* = 0.930). However, SDS scores were negatively correlated with the occupational status of patients (ρ = –0.325, *p* < 0.001), while no significant correlations were observed with BMI (ρ = –0.026, *p* = 0.782) or primary caregiver (ρ = –0.133, *p* = 0.147) (Table 4).

**Table 3.** **Univariate analysis of factors affecting SDS scores in elderly patients with PCa (n = 120)**.

Variables		SDS ($\bar{x}$ ± s)	*t/F*	*p*-value
Age (years)				
	<75	56.65 ± 3.94	0.495	0.622
	≥75	57.06 ± 4.71		
BMI (kg/m^2^)				
	<24	53.44 ± 6.25	1.360	0.176
	≥24	51.27 ± 9.97		
Years of schooling				
	≤6 years	55.41 ± 4.59	2.044	0.134
	6–9 years	57.02 ± 3.95		
	>9 years	55.41 ± 4.59		
Marital status				
	With a spouse	56.87 ± 4.49	0.338	0.736
	Without a spouse	57.25 ± 4.49		
Occupational status				
	Employed	61.06 ± 4.07	4.672	0.011
	Retired	55.54 ± 7.20		
	Other	55.25 ± 4.27		
Average monthly household income (CNY)				
	<3000	59.06 ± 4.57	3.256	0.042
	3001–6000	57.68 ± 4.46		
	>6000	55.00 ± 3.87		
Duration of illness				
	<6 months	55.82 ± 4.24	6.888	0.0015
	6–12 months	56.77 ± 3.88		
	≥12 months	59.33 ± 4.69		
Primary caregiver				
	Spouse	57.73 ± 4.09	13.374	<0.001
	Son or daughter	53.88 ± 3.62		
	Other	59.60 ± 4.79		
Number of chronic diseases				
	0	51.00 ± 4.85	8.745	<0.001
	1	56.02 ± 4.72		
	≥2	58.13 ± 3.71		
Length of hospital stay				
	<15 days	55.64 ± 4.24	1.950	0.054
	≥15 days	57.65 ± 7.16		

Note: SDS, Zung Self-Rating Depression Scale; CNY, Chinese Yuan (1 CNY ≈ 0.14 USD).

**Table 4.** **Correlation analysis of SDS scores in patients with PCa (n = 120)**.

Variables	SDS (ρ)	*p*-value
Age	0.317**	<0.001
BMI	–0.026	0.782
Years of schooling	–0.176	0.055
Marital Status	0.008	0.930
Occupational status	–0.325**	<0.001
Average monthly household income	–0.181*	0.048
Duration of illness	0.266**	0.003
Primary caregiver	–0.133	0.147
Number of chronic diseases	0.280**	0.002
Length of hospital stay	0.204*	0.026

Note: SDS, Zung Self-Rating Depression Scale; ρ, Spearman’s rank correlation coefficient. *: *p* < 0.05; **: *p* < 0.01.

### 3.4 Correlation Analysis of Death Anxiety Status of PCa Patients and Influencing Factors

The results showed that death anxiety scores in PCa patients varied significantly with age, duration of illness, primary caregiver, and number of chronic illnesses (*p* < 0.05) (Table 5). Spearman correlation analysis revealed a positive correlation between death anxiety levels and age (ρ = 0.209, *p* = 0.022), a non-significant positive correlation with duration of illness (ρ = 0.166, *p* = 0.070), and a negative correlation with years of schooling (ρ = –0.210, *p* = 0.021) (Table 6).

**Table 5.** **Univariate analysis of factors affecting T-DAS scores in elderly patients with PCa (n = 120)**.

Variables		T-DAS ($\bar{x}$ ± s)	*t/F*	*p*-value
Age (years)				
	<75	43.52 ± 6.42	3.094	0.003
	≥75	47.76 ± 7.98		
BMI (kg/m^2^)				
	<24	44.84 ± 7.28	0.044	0.965
	≥24	44.77 ± 6.91		
Years of schooling				
	≤6 years	44.71 ± 4.23	2.753	0.068
	6–9 years	46.23 ± 8.30		
	>9 years	42.30 ± 7.90		
Marital status				
	With a spouse	44.93 ± 7.63	0.349	0.728
	Without a spouse	44.30 ± 4.35		
Occupational status				
	Employed	47.69 ± 5.84	1.591	0.208
	Retired	44.49 ± 7.20		
	Other	43.16 ± 8.69		
Average monthly household income (CNY)				
	<3000	44.63 ± 6.68	2.134	0.123
	3001–6000	46.26 ± 7.77		
	>6000	41.62 ± 5.72		
Duration of illness				
	<6 months	44.08 ± 8.04	4.771	0.010
	6–12 months	43.03 ± 4.37		
	≥12 months	48.10 ± 6.73		
Primary caregiver				
	Spouse	46.14 ± 7.72	8.933	<0.001
	Son or daughter	40.53 ± 4.85		
	Other	47.60 ± 4.48		
Number of chronic diseases				
	0	36.00 ± 6.52	4.149	0.018
	1	45.18 ± 7.39		
	≥2	45.23 ± 6.70		
Length of hospital stay				
	<15 days	44.84 ± 7.71	0.029	0.977
	≥15 days	44.80 ± 6.58		

Note: T-DAS, Templer’s Death Anxiety Scale; CNY, Chinese Yuan (1 CNY ≈ 0.14 USD).

**Table 6.** **Correlation analysis of T-DAS scores in patients with PCa (n = 120)**.

Variables	T-DAS (ρ)	*p*-value
Age	0.209*	0.022
BMI	–0.024	0.795
Years of schooling	–0.210*	0.021
Marital status	–0.024	0.793
Occupational status	–0.188*	0.040
Average monthly household income	–0.079	0.390
Duration of illness	0.166	0.070
Primary caregiver	–0.119	0.196
Number of chronic diseases	0.088	0.340
Length of hospital stay	–0.014	0.883

Note: T-DAS, Templer’s Death Anxiety Scale; ρ, Spearman’s rank correlation coefficient. *: *p* < 0.05.

### 3.5 Analysis of Factors Influencing the Quality of Life in PCa Patients and Their Correlations

The results demonstrated significant differences in quality of life scores among PCa patients according to age, BMI, primary caregiver, number of chronic diseases, and marital status (*p* < 0.05) (Table 7). Spearman correlation analysis showed that the quality of life of PCa patients was negatively correlated with age (ρ = –0.436, *p* < 0.001), BMI (ρ = –0.368, *p* < 0.001), and the number of chronic diseases (ρ = –0.255, *p* = 0.005). No significant correlation was observed with the length of hospital stay (ρ = –0.173, *p* = 0.058) (Table 8).

**Table 7.** **Univariate analysis of factors affecting FACT-P scores in elderly patients with PCa (n = 120)**.

Variables		FACT-P ($\bar{x}$ ± s)	*t/F*	*p*-value
Age (years)				
	<75	105.01 ± 5.79	4.213	<0.001
	≥75	100.16 ± 5.90		
BMI (kg/m^2^)				
	<24	104.50 ± 6.36	3.440	<0.001
	≥24	99.96 ± 4.12		
Years of schooling				
	≤6 years	101.78 ± 15.83	0.055	0.947
	6–9 years	101.14 ± 14.63		
	>9 years	100.46 ± 19.29		
Marital status				
	With a spouse	103.62 ± 6.25	0.406	0.686
	Without a spouse	103.62 ± 6.22		
Occupational status				
	Employed	101.63 ± 7.28	1.317	0.272
	Retired	103.63 ± 6.09		
	Other	106.00 ± 4.96		
Average monthly household income (CNY)				
	<3000	104.13 ± 5.61	2.548	0.083
	3001–6000	102.04 ± 6.44		
	>6000	105.90 ± 6.24		
Duration of illness				
	<6 months	104.60 ± 6.33	1.941	0.148
	6–12 months	102.07 ± 5.28		
	≥12 months	102.80 ± 6.65		
Primary caregiver				
	Spouse	103.33 ± 5.73	5.087	0.007
	Son or daughter	105.72 ± 5.19		
	Other	99.73 ± 8.57		
Number of chronic diseases				
	0	112.00 ± 6.08	7.205	0.001
	1	104.33 ± 6.12		
	≥2	102.20 ± 5.74		
Length of hospital stay				
	<15 days	104.58 ± 6.30	1.978	0.050
	≥15 days	102.30 ± 5.95		

Note: FACT-P, Functional Assessment of Cancer Therapy-Prostate; CNY, Chinese Yuan (1 CNY ≈ 0.14 USD).

**Table 8.** **Correlation analysis of FACT-P in patients with PCa (n = 120)**.

Variables	FACT-P (ρ)	*p*-value
Age	–0.436**	<0.001
BMI	–0.368**	<0.001
Years of schooling	0.066	0.477
Marital status	–0.056	0.541
Occupational status	0.144	0.116
Average monthly household income	0.045	0.629
Duration of illness	–0.156	0.089
Primary caregiver	–0.030	0.746
Number of chronic diseases	–0.255**	0.005
Length of hospital stay	–0.173	0.058

Note: FACT-P, Functional Assessment of Cancer Therapy-Prostate; ρ, Spearman’s rank correlation coefficient. **: *p* < 0.01.

### 3.6 Correlation Analysis of SDS, T-DAS, and FACT-P (n = 120)

Spearman correlation analysis showed that depression was negatively correlated with quality of life (ρ = –0.360, *p* < 0.001) in elderly PCa patients, and death anxiety was also negatively correlated with quality of life (ρ = –0.456, *p* < 0.001). Detailed results are presented in Table 9.

**Table 9.** **Correlation analysis of SDS, T-DAS, and FACT-P (ρ, n = 120)**.

Variables	SDS	T-DAS	FACT-P
SDS			
T-DAS	0.493** (*p * < 0.001)		
FACT-P	–0.360** (*p * < 0.001)	–0.456** (*p * < 0.001)	

Note: SDS, Zung Self-Rating Depression Scale; T-DAS, Templer’s Death Anxiety Scale; FACT-P, Functional Assessment of Cancer Therapy-Prostate; ρ, Spearman’s rank correlation coefficient. **: *p* < 0.01.

### 3.7 Mediating Effect of Depression

Model 4 of the PROCESS programme was used for mediation effects analysis, with T-DAS as the independent variable, SDS as the mediator variable, and FACT-P as the dependent variable. The results showed that death anxiety significantly predicted quality of life (β = –0.429, *t* = –6.181, *p* < 0.001) and also significantly predicted depression (β = –0.330, *t* = –6.787, *p* < 0.001). After introducing the mediator, the negative predictive effect of death anxiety on quality of life (β = –0.262, *t* = –3.407, *p* < 0.001) remained significant. The path tests are shown in Table 10, and the decomposition of total, direct, and mediating effects is shown in Table 11. The mediation analysis results showed that the direct effect of T-DAS on FACT-P was significant (β = –0.262, *p* < 0.05). Additionally, the indirect effect of death anxiety on quality of life mediated by depression was also significant (β = –0.167, *p* < 0.05), and the path from the mediator SDS to FACT-P was significant (β = –0.167, *p* < 0.05). These results suggest that depression partially mediates the relationship between death anxiety and quality of life in elderly PCa patients.

**Table 10.** **Mediation analysis of SDS in the relationship between T-DAS and FACT-P (n = 120)**.

Regression Equation		Fitting Index			Coefficient Significance		
Outcome variable	Predictor variable	*R*	*R* ^2^	*F*	β	*t*	*p*-value
FACT-P (Y)	T-DAS (X)	0.495	0.245	38.201	–0.429	–6.181	<0.001
SDS (M)	T-DAS (X)	0.530	0.281	46.062	–0.330	–6.787	<0.001
FACT-P (Y)	T-DAS (X)	0.583	0.340	30.107	–0.262	–3.407	<0.001
	SDS (M)				–0.506	–4.107	<0.001

Note: SDS, Zung Self-Rating Depression Scale; T-DAS, Templer’s Death Anxiety Scale; FACT-P, Functional Assessment of Cancer Therapy-Prostate.

**Table 11.** **Verification of the mediating effects of SDS on the relationship between T-DAS and FACT-P (n = 120)**.

Effect Type	Pathway	Effect	SE	95% CI
Total effect		–0.429	0.069	–0.566~–0.291
Direct effect	T-DAS→FACT-P	–0.262	0.077	–0.414~–0.110
Mediate effect	T-DAS→SDS→FACT-P	–0.167	0.071	–0.331~–0.045

Note: SDS, Zung Self-Rating Depression Scale; T-DAS, Templer’s Death Anxiety Scale; FACT-P, Functional Assessment of Cancer Therapy-Prostate.

## 4. Discussion

PCa is the most common malignant tumor in men and poses a significant threat to the life and health of older men. As the medical paradigm evolves, increasing attention is being directed toward the psychological changes that patients experience during the course of their illness. The literature reports that when patients are diagnosed with cancer, they often experience varying degrees of psychological distress, with this stage representing a peak period for such distress. If not promptly addressed or prevented, psychological distress may persist throughout the entire course of treatment. Depression and anxiety are particularly prevalent among patients experiencing such distress [19, 20].

In the present study, we found that depression scores were significantly higher in patients older than 75 years, those with employed occupational status, those who had been ill for more than six months, those receiving care from others, and those hospitalized for more than 15 days (*p* < 0.05). Depression was also positively correlated with age, duration of illness, and number of chronic diseases while demonstrating a negative correlation with the occupational status of the patient. These findings may be attributed to the fact that as patients age, experience longer durations of illness, accumulate more chronic diseases, and spend more time hospitalized, they are likely to perceive the severity of their condition more acutely, leading to increasing depressive symptoms [21]. Further analysis showed that death anxiety scores varied by age, duration of illness, primary caregiver, and number of chronic illnesses among PCa patients. The level of death anxiety was positively correlated with age and duration of illness but negatively correlated with the level of education. Death anxiety, similar to depression, fluctuates with several influencing factors, such as increasing age. The influence of the primary caregiver on a patient’s death anxiety may arise from prolonged emotional and practical dependence on a spouse, where mutual concern may heighten the intensity of death anxiety [22, 23]. In contrast, children who serve as caregivers for their parents are psychologically more focused on providing “care” and fulfilling the “duty of care” rather than directly confronting mortality, which may make the threat of death less palpable to the patient.

Additionally, individuals with higher education levels generally exhibit greater psychological resilience and possess better resources to manage illness. These individuals often have better access to medical information, an improved understanding of their condition, and more effective coping strategies for anxiety. For instance, they may be more actively involved in treatment decisions or seek psychosocial support interventions to alleviate death anxiety [24]. Similarly, the quality of life among PCa patients was found to be negatively correlated with the patient’s age, BMI, chronic disease type, and primary caregiver.

Moreover, this study identified a mediating role of depressive states in the relationship between death anxiety and quality of life among elderly PCa patients. This finding underscores the importance of addressing depression in managing death anxiety and improving the quality of life in this population. Consistent with previous research, death anxiety is a complex psychological challenge closely linked to depressive mood in cancer patients. Death anxiety typically manifests as an intense fear and worry about dying, along with difficulty accepting the end of life. These feelings often originate from fear of the unknown and a sense of powerlessness due to loss of control of their fate. The diagnosis of cancer itself triggers intense death anxiety, which may be exacerbated by depressive symptoms and can profoundly affect the patient’s emotional, cognitive, and physiological state [25]. For cancer patients, particularly those with progressive disease or ineffective treatment outcomes, prolonged death anxiety can lead to feelings of helplessness and despair, both recognized precursors to depression [26]. Furthermore, death anxiety not only affects psychological well-being but may also aggravate physical symptoms [9]. Depression and anxiety are frequently associated with physical complaints such as insomnia and fatigue, further deteriorating the patient’s overall health. Physical weakness and fatigue exacerbate psychological vulnerability, thereby triggering or worsening depressive symptoms. Additionally, depression distorts patients’ perceptions of negativity, leading them to focus excessively on disease severity and the inevitability of death. It may also impair immune function, leading to increased inflammatory responses and heightened sensitivity to pain [27]. These factors collectively contribute to a stronger perception of death as an imminent threat, thereby exacerbating death anxiety.

As death anxiety increases in patients, it not only alters their emotional state but also has multiple effects on their physical health, cognitive functioning, relationships, and overall quality of life. Death anxiety is typically accompanied by intense feelings of fear, helplessness, and despair, especially when cancer patients face uncertainty about the course of treatment as their disease progresses [27, 28]. This anxiety leads cancer patients to withdraw from social activities and reduce interactions with family and friends. Such social isolation negatively impacts the emotional and psychological well-being of patients and diminishes their access to social support. Social intimacy and support are crucial resources for cancer patients in alleviating the stress of the disease. However, death anxiety makes patients more withdrawn and introverted, thereby creating a vicious cycle [29]. Death anxiety does not only have an impact on a psychological level but also exacerbates the patient’s health condition through physiological responses. Prolonged anxiety and fear can cause physical discomforts, including insomnia, loss of appetite, and body aches and pains. In addition, death anxiety fosters fear and pessimism about the future and a lack of confidence in treatment efficacy and survival prospects. These negative expectations further amplify patients’ sense of helplessness, making it difficult for them to perceive hope for recovery. This mindset may also lead to doubts regarding treatment methods and technologies, ultimately affecting patients’ motivation to continue treatment and impairing doctor-patient relationships [30].

Our study found a mediating effect of depression between death anxiety and quality of life in patients with PCa. Some of the mediating effects identified in this study highlight key areas for nursing interventions in older PCa patients. Depression management should be the central focus of psychological interventions to improve mood and alleviate the psychological distress associated with death anxiety. Pharmacological and psychotherapeutic treatments for depressive symptoms may provide dual benefits by alleviating depression and death anxiety. For example, cognitive behavioral therapy (CBT) and positive psychology interventions can assist patients in identifying and challenging negative thoughts about death, modifying irrational beliefs related to death anxiety, reducing anxiety and depressive symptoms, and encouraging mindfulness to alleviate excessive worry and anxiety. These approaches can improve emotional well-being and enhance overall quality of life [31, 32]. Furthermore, combining psychological support with standard oncology care can promote better treatment outcomes. Personalized interventions that consider the unique emotional and existential challenges faced by older PCa patients may be particularly effective in improving mental health and quality of life. Future research could explore the role of additional psychosocial factors, such as social support and coping strategies, in the relationship between death anxiety, depression, and quality of life. This approach will enable a more comprehensive understanding of the psychological conditions of these patients in clinical settings and ultimately inform the development of more appropriate and effective treatment strategies.

While our study provides valuable insights, there are some limitations. First, the sample size was relatively small, and the geographical scope was limited. Second, self-reported measures were used to assess depression, death anxiety, and quality of life, which may have introduced bias due to individual differences in reporting. Future studies would benefit from incorporating objective measures of physical health and clinician-rated assessments.

## 5. Conclusion

The depressive state, death anxiety, and quality of life of PCa patients are influenced by multiple factors, and a specific correlation exists among these three variables. Death anxiety can directly affect the patient’s quality of life, and it may also contribute to a decline in quality of life through the mediating effect of depression.

## Data Availability

The data used to support the findings of this study are available from the corresponding author upon request.

## Abbreviations

BMI, body mass index; CNY, Chinese Yuan; FACT-P, Functional Assessment of Cancer Therapy-Prostate; PCa, prostate cancer; PCS, PCa subscale; SDS, Self-assessment Scale for Depression; T-DAS, Templer Death Anxiety Scale.
